# How many items from a word list can Alzheimer’s disease patients and normal controls recall? Do they recall in a similar way?

**DOI:** 10.1590/S1980-57642008DN10100009

**Published:** 2007

**Authors:** Marcia Lorena Fagundes Chaves, Ana Luiza Camozzato

**Affiliations:** MD, PhD, Medical Sciences Post-Graduation Course and Neurology Service, Hospital de Clinicas de Porto Alegre, Universidade Federal do Rio Grande do Sul, Porto Alegre, Brazil.

**Keywords:** immediate recall, neuropsychological tests, memory, Alzheimer's disease, elderly, aging, evocação imediata, testes neuropsicológicos,, memória, doença de Alzheimer, idoso, envelhecimento

## Abstract

The serial position effect occurs when individuals are asked to recall a list of information that exceeds normal attention span. Alzheimer's disease (AD) patients show lower scores on word span recall tests when compared to healthy aging subjects, younger individuals or depressed patients.

**Objective:**

To evaluate the immediate free recall and the serial position effect of a 10-word list, emotionally neutral in tone, in Alzheimer's disease (AD) patients and two age-groups of healthy controls.

**Methods:**

The free word recall test was applied in a sample of 44 mild AD outpatients and 168 >50 year and 173 =50 year-old healthy controls. The span of recalled words and order of recollection of each item was recorded. Scores for serial position effect were analyzed.MMSE scores were recorded for all participants. Descriptive statistics and the ANOVA with Tukey test were performed.

**Results:**

The controls scored significantly better than AD patients on the MMSE and word span (p=0.0001). Older controls word span mean ±SD was 5.65±1.75, younger controls was 5.99±1.27, and AD patients was 2.86±1.42. The best recalled item in all groups was the first item of the list. Primacy was observed across the three groups, although AD patients presented lower scores. Recency was diminished among AD patients compared to control groups.

**Conclusions:**

Primacy effect was observed in AD patients as well as in both normal control groups. Recency effect was presented by the normal control groups but was extremely poor among AD patients. The first item was universally best retrieved.

## Abstract (pt)

O efeito de posição de palavras numa lista (posição serial) ocorre quando o indivíduo é solicitado a recuperar informação que excede sua capacidade de atenção. Pacientes com doença de Alzheimer (DA) apresentam escores mais baixos nos testes de recuperação de palavras, quando comparados com idosos sadios ou pacientes com depressão.

**Objetivo:**

Avaliar a evocação imediata livre e o efeito da posição serial de uma lista de 10 palavras, de conteúdo emocional neutro, em pacientes com doença de Alzheimer (DA) e dois grupos etários de controles saudáveis.

**Métodos:**

O teste de evocação livre de palavras foi aplicado em uma amostra de 44 pacientes com DA leve ambulatoriais e 168 controles saudáveis com idade >50 anos e 173 com idade ≤50 anos. O escore da evocação de palavras e a ordem de evocacão de cada item foram registrados. Escores para o efeito da posição seriada foram analisados. O Mini Exame do Estado Mental foi também obtido de todos os participantes. Análise descritiva e ANOVA de uma via com teste de Tukey post hoc foram realizados.

**Resultados:**

Os controles obtiveram escores significativamente mais altos do DA no MEEM e span de palavras (p=0.0001). A extensão de palavras dos controles mais velhos mostrou uma média±DP 5,65±1,75, controls mais jovens foi 5,99±1,27, e pacientes com DA foi 2,86±1,42. O item melhor lembrado em todos os grupos foi o primeiro da lista. O efeito de primazia foi observado nos três grupos, ainda que pacientes com DA tenham apresentado os escores mais baixos. Recência mostrou-se reduzida entre os pacientes em relação aos grupos controles.

**Conclusões:**

O efeito de primazia foi observado nos pacientes com DA bem como nos dois grupos controles normais. O efeito de recência foi apresentado pelos grupos controles normais, mas de forma extremamente empobrecida pelos pacientes com Alzheimer. O primeiro item da lista foi universalmente melhor evocado.

The serial position effect occurs when individuals are asked to recall a list of information that exceeds normal attention span. Normal individuals recall items from a list better which are positioned at the beginning (primacy effect) and the end of a list (recency effect) than those items from the middle of the list.When recall is plotted as a function of serial position, the U-shaped learning curve emerges^1^. Older and younger adults show similar profiles^2-5^ although overall recall in older adults may be lower, with the whole pattern shifted downward. This phenomenon is thought to reflect the concurrent contributions of secondary and primary memory, respectively, to recall performance^6^.

The word span is a common neuropsychological task for the assessment of memory in many conditions such as Alzheimer’s disease^7^. A list of ten unrelated words are orally presented one by one, and subjects are instructed to recall as many items as possible immediately after their presentation (immediate free recall, the traditional span task) and after a predetermined time, in general 5 to 10 minutes (delayed free recall).

These tasks are used worldwide and have been validated in a variety of languages and cultures including Brazilian^7-10^.

Alzheimer’s disease patients show lower scores on word span recall tests when compared to healthy aging subjects, younger individuals or depressed patients^9,11-12^. But do normal elderly individuals and AD patients recall in a similar way? It has previously been demonstrated that AD patients exhibit a significantly reduced primacy effect with a normal recency effect^6^. But what is the clinical or practical application (or meaning) of this information?

With this purpose, the present study evaluated the performance and the serial position effect on the immediate recall of a word list (word span) in Alzheimer’s disease patients and healthy normal controls.

## Methods

For this study, we selected 44 patients with Alzheimer’s disease (AD) from the Alzheimer’s Disease Center and Neurogeriatric Clinic of Hospital de Clinicas de Porto Alegre.We applied the DSM-IV^13^ criteria for dementia and the NINCDS-ADRDA for probable AD^14^. Severity of dementia was classified as mild according to the CDR scale (CDR=1)^15,16^. The diagnosis of dementia was based on clinical history of cognitive and functional impairments and neurological examination. Impairments in cognitive function were documented using standardized psychometric tests. Lewy body dementia, frontotemporal and other rare causes of dementia were also excluded according to standardized criteria^17,18^.

Simultaneously, two normal control groups were selected in different sectors of the hospital (relatives, caregivers and visitors) totaling 341 participants. One hundred and sixty eight older healthy subjects (> 50 years) were included following application of the inclusion and exclusion criteria. Inclusion criteria were functionally independent, cognitively normal. Exclusion criteria were presence of any psychiatric or neurologic disease and use of psychoactive drugs. Younger individuals (=50 years) totaled 173 being selected according to the same eligibility criteria.

All participants were briefly tested for hearing^19^ and vision^20^ with quick screenings (the whispered voice test for screening and the self-reported measures for vision impairment, respectively).

Demographic data of the sample is presented in Table 1.

**Table 1:** Demographic data of sample groups.

	Younger controls	Older controls	Alzheimer's disease	p
	(N=173)	(N=168)	(N=44)	value*
Age (mean SD)	34.81±10.36^a^	65.51±7.57^b^	67.68±6.73^c^	0.001
Education (mean SD)	9.14±4.88^a^	6.22±5.014^b^	6.39±4.05^c^	0.001
Sex - male (N%)	58 (33.5%)	53 (31.5%)	20 (46%)	0.045

* one-way ANOVA; Age: a<b,c (p=0.0001) - post hoc Tukey test; Education: a>b (p=0.001) and a>c (p=0.007) - post hoc Tukey test; Sex: chi-square=3.039; p=0.219.

The sample size was calculated based on the serial position scores effect observed by Foldi et al. (2003)^11^, OR=2.97; % of condition among exposed (AD)=56%; alpha error=5% and beta error=20%. The exposed: nonexposed ratio was 3:1, the number of non-exposed was 123, and the number of exposed was 41.

All participants were assessed by the Mini Mental State Examination (MMSE)^9,21^. Educational attainment was given in years.

The memory task was a 10-item list composed of frequent and concrete words from Brazilian Portuguese, without emotional tone (neutral words), in a simple immediate recall paradigm^8-9^. The order of recollection of each item was recorded. Scores for serial position effect were^11,22^:

***Standard score*** – Standard scores are based on the number of words recalled in a list region divided by the total number of words correctly recalled by the participant.

***Regional score*** – The regional scores were calculated as the number of items recalled divided by the number of items presented from each region of the list.

The regions of the 10-word list were defined as follows:

– First 3 items: primacy region.
– Next 4 words: middle region.
– Last 3 words: recency region.

The study was approved by the Ethics Committee for Medical Research at Hospital de Clinicas de Porto Alegre. Patients and/or their proxies signed an informed consent before being enrolled onto the study.

### Statistical analysis

The statistical analysis was performed using the *Statistical Package for the Social Sciences* (SPSS for MacOs 11.0) software. Descriptive statistics (mean, SD, and frequency) were calculated for demographic data, performance on MMSE and word span. Parametric data were analyzed by one-way ANOVA multivariate procedures. The Chi-square test (with Yates correction or Fisher exact) was used for the association analysis.

## Results

The older control group did not differ from the Alzheimer’s disease patients for age and educational attainment, whereas the younger group showed lower mean age and higher education than both the older control and the dementia group (Table 1). Sex distribution was similar across the three groups.

Younger individuals scored significantly higher than older controls while both scored higher than Alzheimer’s disease patients on the MMSE and word span (Table 2). Young healthy subjects immediately recalled a mean of 6 words out of ten, old normal individuals retrieved around 5.5 words while AD patients recalled a mean of 3 words.

**Table 2:** Word span and Mini Mental State Examination (MMSE; mean±standard deviation): Alzheimer's disease and normal controls.

	Younger controls	Normal controls	Alzheimer's disease	p
	(N=173)	(N=168)	(N=44)	value
Word span	5.99±1.27^a^	5.65±1.75^b^	2.86±1.42^c^	0.0001
MMSE	26.59±3.83^a^	26.31±3.29^b^	18.85±5.56^c^	0.0001

Word list: a>b (p=0.01) and a,b>c (p=0.0001) ) - post hoc Tukey test; Mini Mental: a>b (p=0.008) and a,b>c (p=0.0001)) - post hoc Tukey test.

**Figure 1:**
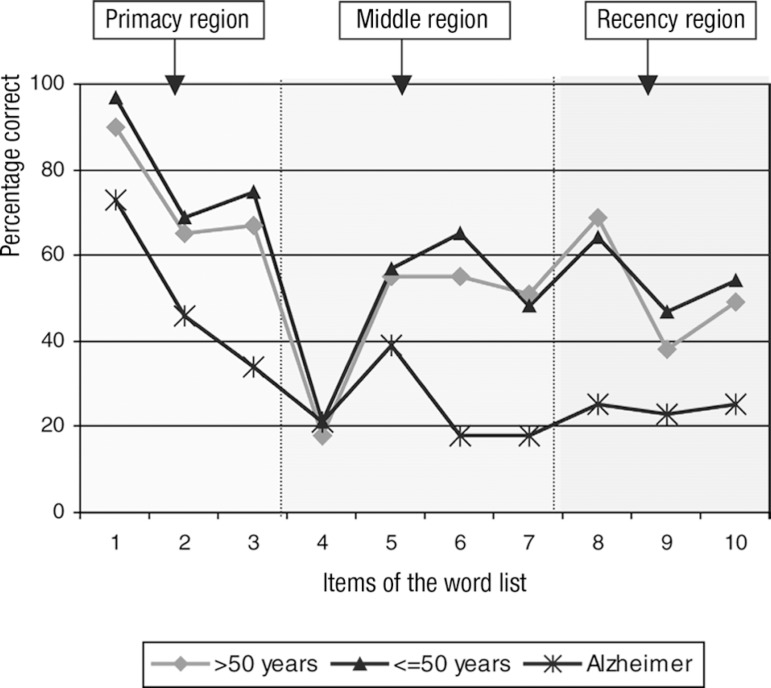
Percentage correct of recollected items from the word list: the serial position effect (primacy, middle and recency) . Alzheimer's disease patients, healthy older (>50 years) and younger (=50 years) controls.

The most recalled item by all groups was the first word of the 10-item list (Figure 1). Among older normal controls, the first word was retrieved by 90% of participants (N=151), and among younger controls by 96.5% (N=167). However, only 73% (N=32) of the Alzheimer’s disease patients recalled the first item of the list. The second word was recalled by 65% (N=109) of older and 69.4% (N=120) of younger controls. Only 45.5% (N=20) of the AD group remembered this item.

The scores of serial position effect were standard and region (Table 3). For primacy, younger and older subjects presented better performances – for region and standard scores – than Alzheimer’s disease patients (p = 0.0001 and p=0.030, respectively). For recency, younger and older subjects presented higher region scores than AD patients (p=0.0001), and younger subjects showed significantly better performance than AD patients on standard scores (p=0.043). For the middle region of the list, the region score presented significant differences among groups (younger and older showed higher scores).

**Table 3:** Distribution of serial position effect among normal controls and Alzheimer's disease patients.

Serial position effect scores	Younger controls (N=173)	Older controls (N=168)	Alzheimer's disease (N=44)
**Primacy**			
Region score	2.80±1.34^a^	2.69±1.30^b^	1.85±1.11^c^
Standard score	0.40±0.15^a^	0.40±0.17^b^	0.48±0.29^c^
**Middle**			
Region score	3.26±1.65^a^	3.04±1.74^b^	2.09±1.44^c^
Standard score	0.31±0.15	0.32±0.18	0.30±0.21
**Recency**			
Region score	3.61±1.83^a^	3.29±1.78^b^	1.38±1.62^c^
Standard score	0.27±0.13^a^	0.27±0.14^b^	0.21±0.24^c^

Primacy - Region score: a,b>c (p=0.0001), Standard score: a,b>c (p=0.030);Middle - Region score: a,b>c (p=0.002); Recency - Region score: a,b>c (p=0.0001), Standard score: a>c (p=0.043).

The unadjusted mean (SD) correct words recalled for each region is presented in Table 4. For all regions, younger and older subjects retrieved more words than Alzheimer’s patients.

**Table 4:** Mean±SD of the score for the items composing the regions of the list (primacy, middle and recency regions).

Region scores	Younger controls (N=173)	Older controls (N=168)	Alzheimer's disease (N=44)
Score for the first 3 items - Primacy region	2.34±0.79^a^	2.18±0.85^b^	1.48±0.90^c^
Score for the 4 middle items - Middle region	1.88±0.99^a^	1.77±0.97^b^	0.95±0.71^c^
Score for the last 3 items - Recency region	1.65±0.79^a^	1.55±0.85^b^	0.70±0.79^c^

First 3 items: a,b>c (p=0.0001);Middle 4 items: a,b>c (p=0.0001); Last 3 items: a,b>c (p=0.0001); Tukey test.

Alzheimer’s disease patients produced a significantly larger proportion of intrusion errors, (32%), followed by younger subjects (16%) (p=0.00001). The older subjects presented the lowest percentage of false recollection (2.4%) (Table 5).

**Table 5:** Distribution of "intrusion" errors (false positive items recalled).

Items of the list	Older controls	Younger controls	AD patients
"False positive" - N (%)	5 (2.4%)	34 (16.2%)	25 (31.8%)
Actual items - N (%)	164 (97.6%)	145 (83.8%)	30 (68.2%)
Total	169	179	55

Chi-square: 36.28; p=0.00001; OR (Mantel-Haenszel) for all strata: 4.89-95% CL 2.7-8.04.

## Discussion

The performance on word span in this immediate free recall paradigm was significantly lower among AD patients than normal controls. The most notable finding was that AD patients from this sample could effectively recall the first three items from the 10-word list, particularly the first word. On the other hand, these patients did not remember the final words of the list as well as expected (recency effect) and as reported in many studies^6,23-27^.

Distinct serial position profiles have been identified in clinical populations. Many studies on Alzheimer’s patients have showed that serial position recall was characterized by a prominent recency effect^6,23-27^. The prominent recency effect could be the result of a rapid decay of information from short-term storage^28^, which in turn prevents item rehearsal or transfer of items to long-term storage, according to a dual storage model^29^.

One study that evaluated patients with major depression alone, major depression with reversible depressionrelated cognitive dysfunction, and primary dementia and major depression has suggested different results^30^. Patients with MD alone acquired significantly more information on the California Verbal Learning Test and showed a more pronounced primacy effect. Item recall of the recency region was equal across the three groups, which was considered surprising by the authors, in that the demented patients did not show the characteristic recency effect.

In the present sample, we observed the primacy effect across groups but not a pronounced recency effect in AD patients. From a practical point of view, if the first words were “kept in mind” the other information was lost. This finding should be taken into account when addressing dementia patients for everyday conversations and delivering information. Using fewer words (just 3 or 4) and stressing them should provide more effective communication. In our culture wordiness is a very common characteristic of communication and it is very difficult for the family members and caregivers to pay attention to the way they communicate with AD patients. To be more effective, we should use fewer words. AD patients can be specifically vulnerable to information overload inherent to a supraspan task, which could be related to the theoretical framework of working memory and to the so-called phonological loop for the temporary storage of acoustic or verbal information as well as the so-called central executive responsible for attentional control^31^.

Alzheimer’s disease patients generated more errors of intrusion than older and younger normal controls, an observation made by previous investigations^30^.We also observed a significant percentage of intrusions among younger controls. Younger adults can show more subjective organization than older individuals^32^, which could lead to occurrence of this proportion of memory errors.

Primacy and recency effects are currently believed to reflect the temporal distinctiveness of individual items in memory representations^33-35^.When the time interval between presentation of the list and memory testing is increased, the serial position curve changes from a predominantly recency to a predominantly primacy type function^35-38^, with cross over on different time scales for pigeons, monkeys and humans^38^. In a study with pigeons, monkeys and humans, the task for all three species was a serial-probe-recognition task^38^. The trials consisted of pressing down a three-position T lever (monkeys and humans) or pecking on a 9 by 9.3 cm clear window (pigeons). Lists of color slides were projected one at a time on the upper of two screens. A probe item was projected on the lower screen after a delay (retention interval) from the last list item. If the probe item was a repeat of one of the list items (“same” trial), a correct response by humans or monkeys was a lever movement to the right and by pigeons a peck to a right disk (lighted red). Otherwise (on “different” trials) a left lever movement or a left disk (lighted green) peck was correct. The authors suggested that qualitative similarity implies similar memory mechanisms. This suggests that changes in serial position curves with retention interval may reflect the temporal organization of information processing in short-term memory. The need remains for more information about cultural differences in serial position effect, because this could be one explanation for the differential finding of pronounced primacy and poor recency effect in AD patients. Nevertheless, further examination of the serial position effect and its relationship to other aspects of culture is clearly warranted.
